# Removal Modeling and Experimental Verification of Magnetorheological Polishing Fused Silica Glass

**DOI:** 10.3390/mi14010054

**Published:** 2022-12-25

**Authors:** Limin Zhang, Weixing Li, Jiakang Zhou, Mingming Lu, Qiang Liu, Yongsheng Du, Yakun Yang

**Affiliations:** 1 Jilin Provincial International Cooperation Key Laboratory for High-Performance Manufacturing and Testing, School of Mechatronic Engineering, Changchun University of Technology, Changchun 130012, China; 2 School of Machinery and Automation, Weifang University, Weifang 261061, China; 3 Key Laboratory of CNC Equipment Reliability, Ministry of Education, Jilin University, Changchun 130012, China

**Keywords:** magnetorheological polishing, removal modeling, dwell time, surface precision

## Abstract

Compared to conventional polishing methods, magnetorheological polishing has no subsurface damage and a has good polishing effect, which is suitable for fused silica glass surface processing. However, the existing magnetorheological polishing material removal model has low processing efficiency and uneven removal, which cannot realize the deterministic processing of parts. The material removal (MR) model of fused silica glass is established by convolving the dwell time with the material removal function. The residence time is Fourier transformed. The consequence of process variable such as machining time, workpiece rotational frequency, machining gap and X-direction deflection on the MR of workpiece interface are analyzed. Experiments verify the validity of the material removal model. The surface precision PV value of the workpiece surface under the optimal process parameters was decreased from 7.959 nm to 0.609 nm for machining. The experiment results indicate that the established MR model can be implemented as the deterministic MR of the optical surface and ameliorate the surface accuracy of the workpiece surface.

## 1. Introduction

Fused silica glass has the characteristics of optical transparency, space stability, high hardness, high surface finish and good thermal stability. It is widely used in aerospace, optical systems, electronic devices, defense technology and other fields [1,2,3]. Fused silica glass is a classic hard-to-cut material. Because of its high rigidity, brittleness and low ductility, it is easy to produce cracks and chips during processing, which seriously affects the surface quality of the processed parts [4,5]. Various fields have required higher demands of the processing precision, quality and decrease of fused silica glass [6,7]. The study of the surface removal model of the fused silica glass is crucial to ameliorating the machining precision and quality and to decreasing fused silica glass.

Compared to conventional polishing methods, magnetorheological polishing has no subsurface damage and has a good polishing effect, which is suitable for the surface processing of fused silica glass [8,9]. A new magnetorheological polishing technique was applied. The magnetorheological effect generated by magnetorheological fluid under the action of a magnetic field has been used to restrict and clamp the abrasive material so that the abrasive particles are in a semi-fixed state and optical surface polishing is carried out [10,11,12]. The establishment of a suitable material removal (MR) model in the magnetorheological polishing process is the basis for achieving deterministic processing. Therefore, many domestic scholars have done a great deal of research in material removal in magnetorheological polishing process. Pan et al. [13] investigated the consequence of various process variables on the workpiece surface polishing pressure based on the hydrodynamics and the Preston equation and found a material removal rate (MRR) model for cluster magnetic rheological polishing under a dynamic magnetic field. Wang et al. [14] founded the MRR model of reciprocating magnetorheological polishing (RMRP) based on the RMRP principle and the Preston equation. Liu et al. [12] presented a new MRR co-model, including shear stress and pressure, which experimentally verified the validity of the MRR model in the different process variables.

Tian et al. [15] established a hydrodynamic model in the polishing area, solved the MR parameters and theoretically researched the influence of magnetorheological fluid (MRF) temperature on the MRR. Ming et al. [16] proposed an effective non-Newton fluid polishing method, studied the removal and evolution mechanism of nanometer-scale materials on the zirconium ceramic surface of zirconium ceramics and founded a prognostication model of the MRR. Zhai et al. [17] established an MRR model based on the Preston equation, which predicted the MRR distribution and the mean removal rate depth on a polished sapphire surface. Yao et al. [18] presented an MR model for studying the MR and roughness of the surface for soft cushion and free abrasive cylinder polishing from both theoretical and experimental aspects.

The Fourier transform is based on hydrodynamics, and the Preston equation was applied to the residence time. The transformed residence time is convolved with the material removal function to establish a fused silica glass material removal model. The influence of processing time, workpiece rotational frequency, machining clearance and X-direction deflection process variable on the workpiece surface MR is analyzed and the availability of the established MR model is validated.

## 2. Modeling of Magnetorheological Polishing Removal

The magnetorheological polishing effect generated by magnetorheological polishing forms a layer of Bingham body polishing pad on the fused silica glass workpiece surface, which produces polishing force and relative sliding speed on the workpiece surface, realizing the efficient removal of polishing surface materials. By combining an extended version of the Preston equation with the mechanical properties of actual polishing [19,20], the material removal function is proposed:

(1) $$MRR=Kn\frac{P_{i}^{3/4}}{L_{m,c}}\frac{E^{5/4}}{k_{c}H_{V}^{2}}PV$$

where *P_i_*, *P* is the average load borne by individual particles and the resultant force of the workpiece surface, and n is the amount of entry dots. *Lm*, *c* is the mean particle diameter of loaded particles, and *k* is the Preston coefficient. *E*, *kc* and *H_V_* are Young’s modulus, the workpiece’s fracture ductility and the workpiece’s rigidity, respectively. The *MRR* positively correlates with the pressure *P* on the surface of the workpiece material and the relative velocity *V* between the workpiece surface and the polishing disc. It is also proportional to the number of entry points n and the standard load *P_i_* of a single particle. The *MRR* negatively correlates with the loaded particles’ mean particle diameter. *Lm*, *c*, *E*, *kc* and *H_V_* are constants related to the material’s properties.

### 2.1. Solutions of Pressure P

In the magnetorheological polishing process, the force imposed by the MRF on the surface of the fused silica glass workpiece can consist of three parts: the fluid pressure *P_d_* comes into being in the magnetorheological fluid on the fused silica glass workpiece surface, the pressure *P_m_* is exerted by the gradient magnetic field on the fused silica glass workpiece surface and the interrelation force *P_Z_* is the force between the magnetic micro-particle. The resultant force *P* of the workpiece surface can be represented as:

(2) $$P=P_{d}+P_{m}+P_{z}$$

The hydrodynamic pressure *P_d_* comes into being in the magnetorheological fluid on the fused silica glass workpiece surface:

(3) $$P_{d}=\frac{6v\eta_{N}\left[1+\frac{\eta_{t}}{\eta_{N}}\frac{\left|v\right|}{h_{e}}\right]\left(h_{e}-h_{\min}\right)l}{h_{e}^{3}}$$

where *η*, *η_N_*, *η_t_* and *v* are the viscidity of magnetic fluid, a Newtonian fluid’s viscosity coefficient, the pseudo-plastic fluid’s viscosity and the relative velocity between the flat plate and polishing disc, respectively. The *l* and *h*_min_ are the width and the minimum fluid membrane thickness, respectively. The *h_e_* is the fluid membrane thickness at the narrow opening in the fluid dynamic principle.

The pressure *P_m_* exerted by the gradient magnetic field on the fused silica glass workpiece surface:

(4) $$P_{m}=\int_{0}^{H}M_{f}dH=\phi \int_{0}^{H}MdH=\frac{3\phi \mu_{0}\mu_{p}}{2}\cdot \frac{\mu_{1}-\mu_{p}}{\mu_{1}+2\mu_{p}}\cdot {\left(\frac{B/\mu_{0}}{1+3\mu_{0}\mu_{p}\frac{\mu_{1}-\mu_{p}}{\mu_{1}+2\mu_{p}}}\right)}^{2}$$

where *μ*_0_, *μ_p_* and *μ*_1_ are vacuum permeability, the permeability of base liquid and the magnetic permeability of magnetic materials, respectively. The *H* and *M* are the magnetic field strength on the fused silica glass workpiece surface and the magnetization of hydroxyl iron powder. *ϕ* is the volume ratio of magnetic particles.

The interaction force *P_Z_* between the magnetic particles:

(5) $$P_{Z}=\sum_{i=1}^{N_{CIP}}P_{Z-x_{i}}\sin\theta_{1}+\sum_{i=1}^{N_{CIP}}P_{Z-y_{i}}\cos\theta_{1}$$

In the formula, $P_{Z-x_{i}}$, $P_{Z-y_{i}}$ are the inter-particle interaction component of hydroxyl iron powder, respectively; *θ*_1_ is the angle between the line of the hydroxyl iron powder magnetic particle and the X-axis of the XOY system of coordinates.

### 2.2. Solution of the Relative Speed

**Figure 1:**
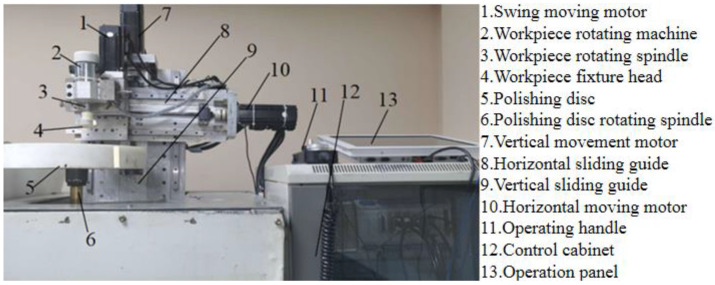
Magnetorheological polishing device.

The relative speed between the workpiece surface and the throwing disc has three velocity components. The calculation model of its components is shown in Figure 1. These three speed components are the rotational speed v1 of the workpiece, the rotation speed of the polishing disc v2 and the feed speed v3 of the workpiece along the Y-axis of the machine tool. The magnetic polar is embedded in the polishing disc, so the magnetorheological polishing head’s rotation speed generated under the magnetic pole’s action moves synchronously with the polishing plate disc. The rotation velocity of the polishing disc is the rotation speed of the polishing tool head.

Rotation speed of the workpiece *v*_1_:

(6) $$v_{1}=2\pi \omega r$$

*ω* is the rotational speed of the workpiece, and *r* is the interval from the rotation center of the workpiece to the polishing dot.

The rotation speed of the polishing disc *v*_2_:

(7) $$v_{2}=2\pi \omega_{1}r_{1}$$

*ω*_1_ is the speed of the polishing disc, and *r*_1_ the radius of the magnetorheological polishing head.

(8) $$v=\sqrt{{\left(2\pi \omega r\right)}^{2}+{\left(v_{3}+2\pi \omega_{1}r_{1}\right)}^{2}}$$

### 2.3. Solution of the Dwell Time

The surface shape of the workpiece surface changes after actual machining and the amount of material removed △*R*(*x*, *y*) can be confirmed by calculating the difference between the workpiece surface before and after processing. The dwell time can be calculated from the material removal function.

(9) ΔR(x,y)=R(x,y)∗T(x,y)

In the formula, $R\left(x,y\right)=\frac{d\Delta R\left(x,y\right)}{dt}$, *T*(*x*, *y*) is the resident time.

A Fourier transform on the dwell time can be obtained:

(10) $$R\left(\omega_{x},\omega_{y}\right)=T\left(\omega_{x},\omega_{y}\right)\cdot G\left(\omega_{x},\omega_{y}\right)$$

In the formula, *R*(*ω_x_*, *ω_y_*), *T*(*ω_x_*, *ω_y_*), *G*(*ω_x_*, *ω_y_*) are the Fourier transform forms of △*R*(*x*, *y*), *T*(*x*, *y*), *R*(*x*, *y*).

(11) $$T\left(\omega_{x},\omega_{y}\right)=R\left(\omega_{x},\omega_{y}\right)/G\left(\omega_{x},\omega_{y}\right)$$

A Fourier transform of *T*(*ω_x_*, *ω_y_*) again yields the function of resident time. At the same time, in the calculation process, if there is a negative time value, directly assigning the negative value to 0 will produce a significant error. A dwell removal factor △*T*(*ω_x_*, *ω_y_*) is introduced to remove this error.

(12) $$\Delta T\left(\omega_{x},\omega_{y}\right)=\frac{\left|G\left(\omega_{x},\omega_{y}\right)\right|}{\alpha {\left|G\left(0,0\right)\right|}^{2}+{\left|G\left(\omega_{x},\omega_{y}\right)\right|}^{2}}$$

*α* value is the coefficient, which can be set. Repeat the above equation calculation until the error does not meet the requirements.

The removal function and residence time are convoluted further to improve the machining precision of the removal function model. The convolution formula is as follows:

(13) $$MRR_{a}=\left(Kn\frac{P_{i}^{3/4}}{L_{m,c}}\frac{E^{5/4}}{k_{c}H_{V}^{2}}PV\right)⊗T\left(x,y\right)$$

## 3. Experimental Research

Four sets of magnetorheological polishing experiments were carried out on the fused silica glass workpiece surface to verify the material removal model’s certainty and validity. The workpiece parameters were a diameter of 27.5 mm and a thickness of 15 mm. Process parameters of the experiment were processing time, workpiece rotational frequency, processing gap and X-direction deflection. The values of the process variable are shown in Table 1. The laboratory designed the magnetorheological polishing device, and the magnetorheological polishing fluid was also configured by itself based on previous research. The magnetorheological finishing liquid used in the experiment was constituted of hydroxyl iron powder with a diameter of 2.5 μm, cerium oxide powder with a diameter of 10 μm, deionized water and a stabilizer. The main component of the stabilizer was sodium dodecyl sulfonate. The main components of the magnetorheological finishing liquid are shown in Table 2. The magnetorheological polishing device [21] is shown in Figure 1.

## 4. Results and Analysis

**Figure 2:**
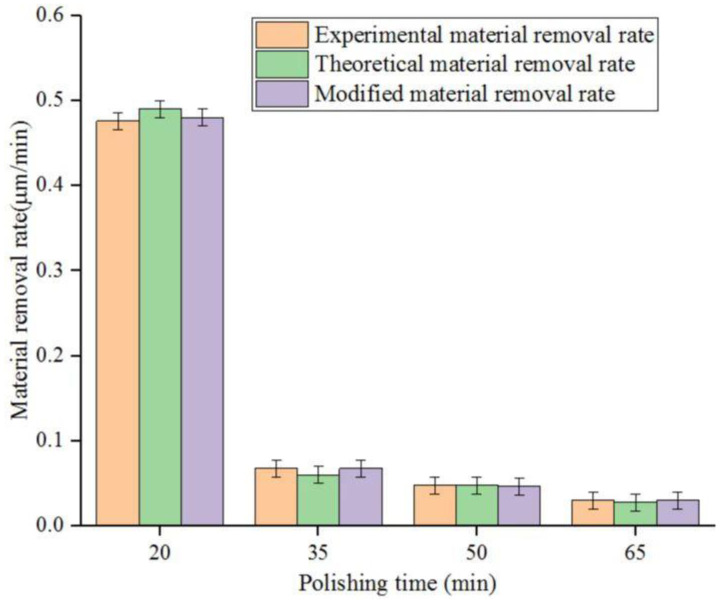
Three mean MRRs of polished workpiece surface at different polishing times.

In the material removal bar chart in Figure 2, the orange, green and purple strips represent the average theoretical material removal, average experimental material removal and average modified material removal at each point of polishing time of 20 min, 35 min, 50 min and 65 min, respectively. The material removal rate of fused silica glass was inversely proportional to the polishing time. With the increase in that polishing time, as seen in Figure 2, the mean theoretical material removal, average experimental material removal and average corrected material removal all displayed a downward trend. The average material removal rate of the workpiece was maximum (0.476335 μm/min) when the polishing duration was 20 min, and the average MRR was the most petite (0.0278455 μm/min) when the polishing was 65 min. The mean MRR at 20–35 min decreased faster than at 35–65 min. From the overall trend, the corrected mean material removal was closer to the mean actual removal.

**Figure 3:**
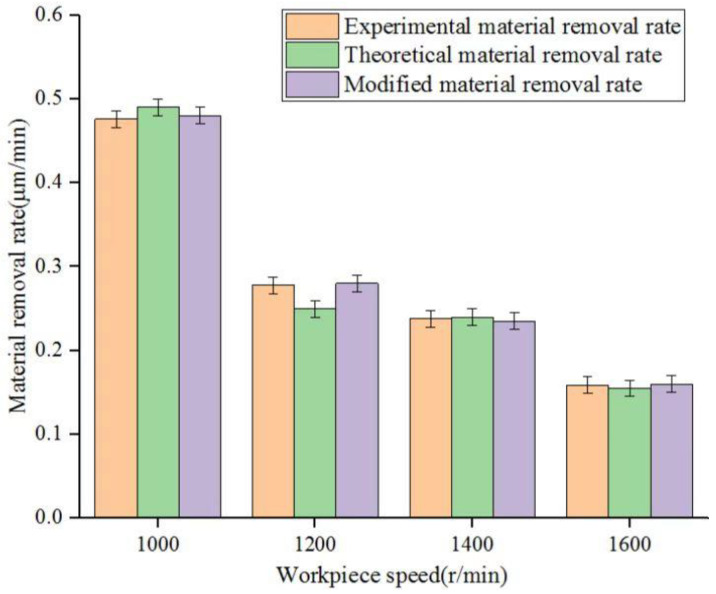
Three mean MRRs of polished workpiece surface at different workpiece speeds.

In the strip chart of material removal in Figure 3, the orange, green and purple strips represent the average theoretical material removal, average experimental material removal and average corrected material removal at each point when the workpiece rotational frequency is 1000 r/min, 1200 r/min, 1400 r/min and 1600 r/min, respectively. As the rotation speed of the workpiece increases, the interaction time between the corresponding micro-polished grinding heads and the surface of the fused silica glass workpiece decreases accordingly. As seen in Figure 3, the average theoretical material removal, average experimental material removal and average modified material removal all displayed a downward trend. As the rotation speed of the workpiece continued to increase beyond the limit, most of the flux linkage formed in the polished area broke and the abrasive particles clamped by the flux linkage reduced the material removal effect on the workpiece surface. Compared with the consequence of polishing time on the average MRR, the overall downward trend is slower, and the modified average material removal is closer to the average actual removal.

**Figure 4:**
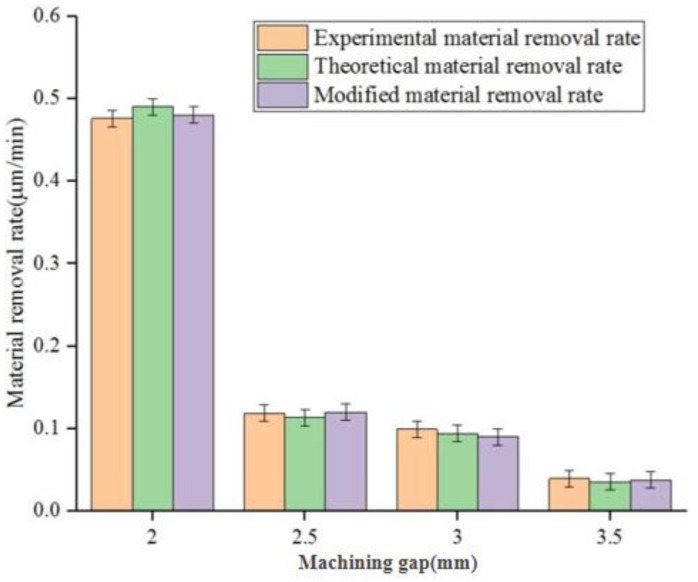
Three mean MRRs of polished workpiece surface at different machining gaps.

In the material removal bar chart in Figure 4, the orange, green and purple strips represent the average theoretical material removal, average experimental material removal and average corrected material removal at each point of 2 mm, 2.5 mm, 3 mm and 3.5 mm machining gaps, respectively. With an increase of the machining gap, the average load between the abrasive particle and the workpiece surface decreased, the contact stress between the polishing pad and the workpiece surface increased and the repair ability of the MR polishing pad was enhanced. As the machining gap continued to increase, the average load acting on the surface of the fused silica glass workpiece by a single tiny polishing head continued to decrease. When the machining gap between the workpiece and the polishing pad was too large, the average load between the magnetorheological polishing pad and the workpiece was minimal. It is difficult to remove the machining traces left by the previous process. Therefore, the mean theoretical MR, average experimental material removal and average modified material removal all displayed a downward trend. What was consistent was with the influence of polishing time and workpiece rotational velocity on the average MR. When the polishing gap was 2 mm, the mean MRR of the workpiece was the largest (0.476335 μm/min), and the average MRR was smallest (0.0992362 μm/min) at 3.5 mm. The average material removal rate at 2–2.5 mm was faster than at 2.5–3 mm and 3–3.5 mm. The average material removal rate at 2.5–3 mm was slower than at 3–3.5 mm. From the overall trend, the modified average material removal was closer to the average actual removal.

**Figure 5:**
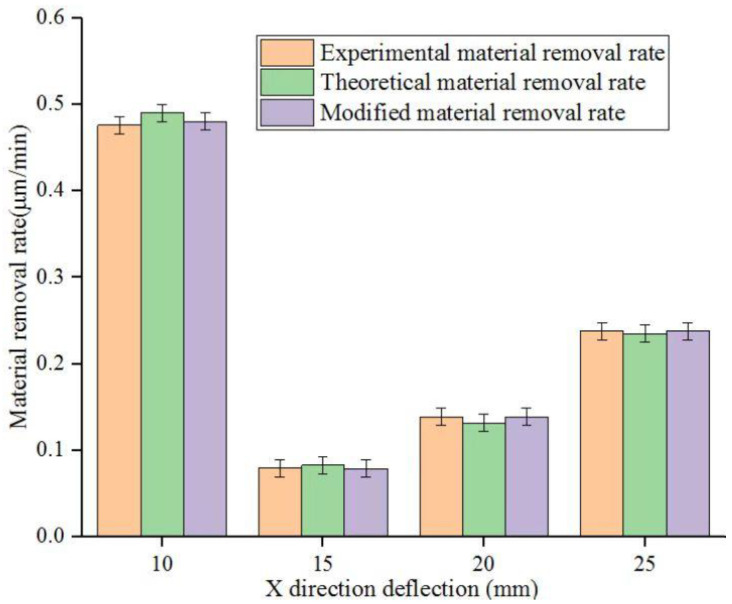
Three mean MRRs of polished workpiece surface at different X-direction deflections.

In the strip chart of material removal in Figure 5, the orange, green and purple strips represent the average theoretical material removal, average experimental material removal and average corrected material removal at each point when the X deflection is 10 mm, 15 mm, 20 mm and 25 mm, respectively. The average experimental material removal was 0.476335 μm/min, 0.2381668 μm/min, 0.0793889 μm/min and 0.1389306 μm/min, respectively. The average theoretical material removal, the average experimental and the average corrected material removal decreased first and then increased with an increase of X-direction deflection. As the X-direction deflection distance increases, the polishing path becomes longer. The frequency of cerium oxide (Ce_2_O) abrasive particles passing through the surface of fused silica glass workpiece decreases, resulting in the gradual decrease of material removal rate with an increase of X-direction deflection distance. At the same time, the workpiece deflection can compensate the machining area of the workpiece to a certain extent so that when the deflection amplitude increases, the machining uniformity gradually improves. It differs from the influence of polishing time, machining gap and workpiece rotational frequency on the mean MRR. However, the corrected average material removal trend is more accessible than the average actual removal.

**Figure 6:**
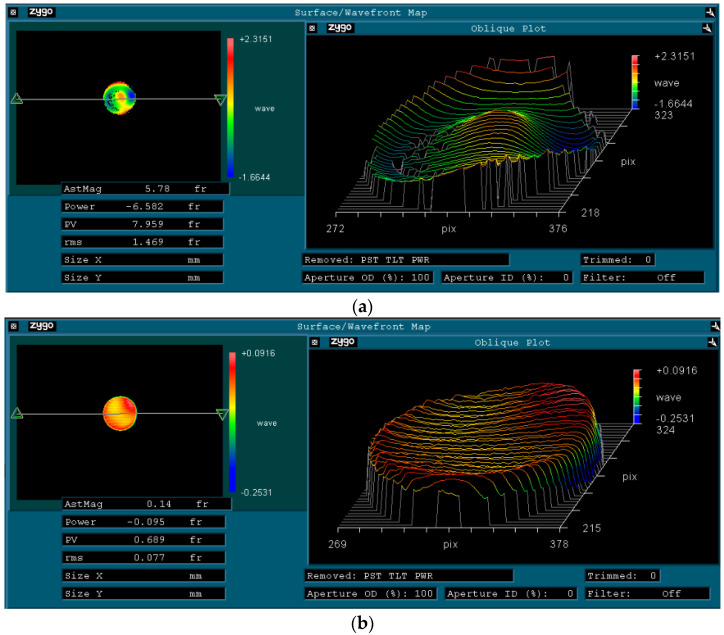
Surface (Wave-front map) of fused silica glass pre- and post-processing: (**a**) pre-processing; (**b**) post-processing.

The optimal process parameters determined by the single factor experiment was used to polish fused silica glass again. The experiment determines the MR amount and MRR before and after processing. The average material removal rate obtained from the experiment contrasts the average theoretical MRR and the average modified MRR. The results show that the modified average MRR was closer to the average actual removal rate. The surface morphology of the workpiece surface pre- and post-processing is demonstrated in Figure 6. It can be seen from Figure 6 that the accuracy of the surface (wave-front map) of the workpiece surface was significantly improved under the optimal process parameters. The surface precision PV value of the workpiece surface was decreased from 7.959 nm to 0.609 nm for machining.

## 5. Conclusions

The Fourier transform based on hydrodynamics and the Preston equation was applied to the residence time and then the transformed residence time was convolved with the MR function. The consequence of process variables such as processing time, workpiece rotational frequency, machining gap and X-direction deflection on the MR of the workpiece surface were analyzed and the validity of the material removal model was verified. Compared with the previous MR models, the established MR model realized the workpiece surface’s deterministic processing and improved the workpiece surface’s surface accuracy. Under the optimal process parameters, the experimental average MRR of the workpiece surface was closer to the average corrected MRR, and the surface shape error of the workpiece converged. The surface precision PV value of the workpiece surface was decreased from 7.959 nm to 0.609 nm for machining.

## Figures and Tables

**Table 1:** Magnetorheological polishing process parameters and values.

Process Parameters	Value Range
Processing time (min)	20–65
Workpiece rotational frequency (r/min)	1000–1600
Machining gap (mm)	2–3.5
X-direction deflection (mm)	10–25

**Table 2:** Composition of the magnetorheological finishing liquid.

Materials	Percentage (vol%)
Hydroxyl iron powder	35
Deionized water	55
Ce_2_O	2–3.5
Stabilizer	10–25

## Data Availability

Not applicable.
